# Partial Sets of Artificial Teeth

**Published:** 1857-09

**Authors:** H. R. Smith


### PARTIAL SETS OF ARTIFICIAL TEETH.

BY H. R. SMITH.

In my last communication I furnished the outlines of inserting full sets of teeth by suction or atmospheric plates. And if the student is sufficiently careful in carrying out the directions, he can not, with a fair amount of skill, fail to give his patient a good operation.

There are other mechanical operations much more common to the young practitioner than full sets, and those that will test his skill even more than full sets, namely, partial sets. In full sets, a few general rules are all that is necessary to be observed to insure success, while with partial sets the circumstances attending each case have more or less peculiarities, and call for a greater degree of skill. But few partial sets succeed according to the wishes of the patient, or even the operator. But to my mind, for the most part it can be traced either to the unskillfulness or neglect of the operator. Perhaps I may be considered as giving too free use of my words when I say this, but nevertheless I believe it to be a fact, and in doing so, I will proceed to state some of the causes of failure.

In the first place, dentists are not generally sufficiently well posted in physiology and pathology to know what is a true healthy state of the mouth and teeth, and the variations from a state of health. Hence, being ignorant of these things, it is not to be expected that they can succeed in making their mechanical operations successful.

The surgical mechanic would not think of affixing an artificial limb to an imperfectly healed or diseased stump after amputation, then why should the dentist expect to succeed under similar circumstances?

It is true, the patient may tolerate a fixture in a diseased mouth, but it will never become fully satisfactory. Then here, let me say, is the most general and prominent cause of all those bad operations which daily come to our notice.

The next cause is the imperfect adaptation of the fixture. It is not sufficient that the piece shall be neatly made and highly finished, although these are essential qualities in the job; but the adaptation to the mouth must be good, or the fixture will be useful only as "show work" to adorn our parlor table, or draw custom from the crowd who demand to see a specimen of our skill, which, as all sensible persons will be aware, does not entirely fill the "bill" which we have proposed.

Then the first indications are a critical and full examination and a correct diagnosis. The causes which have operated to produce disease and decay should be investigated, the effects produced by such disease, and the final result. Certain and absolute effects must follow, in the course of certain diseases, unless by some means we can arrest the cause. When this can be done, we have only to repair the ravages already made, and to avoid the reappearance of the former causes.

When the above is well understood, it will be very easy to succeed in carrying out the indications.

It is not always possible to do what our skill and judgment would demand; when such is the case, the patient should be given to understand that they can not receive, under the circumstances, our best operations. Oftentimes our patients are not able to meet the demand necessary to insure them the best operation. When this is the case, we have one of three courses to pursue: either refuse to operate—to do the job, and share the profits with the patient—or to do a job within the means of the patient. The first may sometimes be best, the second, when we can afford it, always proper, and the third often necessary, and will, while remunerating us, satisfy the patient.

In a former article I gave directions for preparation of the mouth for artificial fixtures, both for full and partial sets.

Then, for partial sets, the first thing is to decide how we shall retain the job in the mouth.

My first preference is, of course, for a suction plate; but unless the plate is made large, it will not be sufficiently firm in the mouth to use for mastication, which all will acknowledge to be one, if not the greatest indication.

For instance, a single central incisor is to be inserted with a plate the size of a dime or a quarter of a dollar. With the best adaptation possible, it could not be well used to masticate with, while twice or thrice the size would make it so.

If the patient can bear the expense, all well, but if not, then we must adopt some other way to insure the best operation for his means.

The only precautions to be taken in making a partial suction plate are those to make an entire piece, namely, a perfect fit, and when admissible, a chamber. The edges of the plate should be perfectly fitted around the necks of the teeth and other parts of the arch, or the air will insinuate itself under, and destroy the suction.

If there is any hard ridge through the median line of the arch, the plate should be relieved from it, or it will rock, and thereby destroy the suction.

Another method is a very good one where there are well defined necks to the remaining teeth, namely, tubes soldered to the plate on each side, so as to bear against the necks of the teeth on each side of the mouth in an antagonizing position. They are made by making a tube about one-eighth of an inch or a little longer, and about one-tenth of an inch in diameter, closed at one end. This is soldered to the plate where the open end will point towards the neck of a tooth, and lay as nearly parallel with the necks of the teeth as possible. Into this is inserted a hickory pivot, which, projecting out of the tube a little, will bear against the neck of the natural tooth.

When the teeth are sensitive, this method has a great advantage over clasps, or any other, when the plate comes in contact with the teeth, as little or no galvanic action can be carried on between the plate and teeth, as the wood is a bad conductor of galvanism.

Another advantage of the tubes is this, we do not have to separate with the file in order to fasten our attachments to sustain the plate. These pivots have to be removed occasionally, but with a little instruction from the dentist, the patient will be able to prepare and adjust them, whenever necessary, for himself.

The next method is by partial or stay clasps, which act in a similar way to the pivot and tube, excepting that it brings the plate in contact with the necks of the teeth.

They are made by fitting stays or partial clasps to go as far around the inner neck of the tooth as possible, without passing between the teeth.

By putting one or more on each side of the plate, and fitting them accurately to the neck of the tooth, will sustain a plate quite perfectly. These stays should, however, be as wide as the tooth will permit of, or the plate will soon have a rocking motion, and that motion would soon wear a groove around the neck of the tooth, thereby causing its destruction.

The next method is when we use entire clasps.

This method has of late been the subject of much controversy, and been by many entirely discountenanced. But I am sorry to say it, many of those who abuse it in public, resort to it very frequently in practice.

But like calomel in medicine, it has gone into partial disuse, because the method has been so much abused in practice.

By the quack, calomel is used, because he does not know how to use any thing else; therefore he often uses it very improperly.

So it is with clasps in dentistry, men resort to them when they cannot retain their plates in any other way, for they can make their plates stay in the mouth when there is no adaptation. Their jobs are what might be called "plyer work," as most of the fit is made by bending with plyers. Such work must of course cause destruction to the natural teeth.

Another cause for the destruction of the clasp teeth is from an ignorance of the pathological condition of the mouth and teeth.

There are certain indications which should entirely forbid the use of clasps. First, they should never be used on young persons until the teeth are fully organized. Ossification not being completed at the points of the roots and the process yet spongy, the teeth are liable to be loosened in the alveola, and irritation and displacement result. At this stage decay is more liable to attack the teeth, and is more rapid in its course. Also, there being a higher state of organization, chemical and galvanic action acts with greater force.

Secondly, clasps should not be used when a high state of acidity exists in the mouth, for galvanic action is sure under such circumstances to cause destruction to the natural teeth.

But when the teeth are well organized, of a firm, healthy texture, and with alkaline secretions very little objection can be had to clasps.

As a majority of the clasp jobs fail from a want of adaptation, it is to that point we should look for a remedy. I will give a few general directions which should always be observed.

In the first place the plate should be perfectly fitted to the mouth, or well fitted around the necks of the teeth, but should not touch them.

The clasps should be perfectly fitted around the teeth and as wide as the teeth will permit, and set without putting the teeth on a strain.

As inferior gold creates galvanic action, very fine gold should be used, and fine solder.

The work should be done with the greatest care, for it requires greater skill to do a good clasp job, and one that will succeed perfectly, than any other.

And then to ensure success with all partial jobs, the patient should be well advised in relation to keeping the mouth and remaining teeth perfectly cleansed, for when it is not done, food and foreign matter collects around the clasps and teeth, and decay and chemical action will take place, and as a matter of course the teeth must decay, and as a result the poor clasps come in for the blame which should be only laid to the negligence of the patient, or unskilfulness of the operator.

